# Characterization of Human Mesenchymal Stem Cells from Ewing Sarcoma Patients. Pathogenetic Implications

**DOI:** 10.1371/journal.pone.0085814

**Published:** 2014-02-03

**Authors:** Ana Teresa Amaral, Maria Cristina Manara, Dagmar Berghuis, José Luis Ordóñez, Michele Biscuola, Maria Angeles Lopez-García, Daniel Osuna, Enrico Lucarelli, Francesco Alviano, Arjan Lankester, Katia Scotlandi, Enrique de Álava

**Affiliations:** 1 Molecular Pathology Program, Institute of Biomedical Research of Salamanca-Centro de Investigación del Cáncer, Centro de Investigación del Cáncer (IBSAL-CIC), Salamanca, Spain; 2 CRS Sviluppo di Terapie Biomolecolari, Oncologia Sperimentale, Istituto Ortopedico Rizzoli (IOR), Bologna, Italy; 3 Department of Pediatrics and Biobank, Leiden University Medical Center (LUMC), Leiden, The Netherlands; 4 Osteoarticolar Regeneration Laboratory, Istituto Ortopedico Rizzoli (IOR), Bologna, Italy; 5 Dipartimento di Istologia, Embriologia e Biologia, Istituto Ortopedico Rizzoli (IOR), Bologna, Italy; 6 Instituto de Biomedicina de Sevilla (IBiS), CSIC-Universidad de Sevilla, Department of Pathology and Biobank, Hospital Universitario Virgen del Rocío, Seville, Spain; Johns Hopkins University, United States of America

## Abstract

**Background:**

Ewing Sarcoma (EWS) is a mesenchymal-derived tumor that generally arises in bone and soft tissue. Intensive research regarding the pathogenesis of EWS has been insufficient to pinpoint the early events of Ewing sarcomagenesis. However, the Mesenchymal Stem Cell (MSC) is currently accepted as the most probable cell of origin.

**Materials and Methods:**

In an initial study regarding a deep characterization of MSC obtained specifically from EWS patients (MSC-P), we compared them with MSC derived from healthy donors (MSC-HD) and EWS cell lines. We evaluated the presence of the EWS-FLI1 gene fusion and EWSR1 gene rearrangements in MSC-P. The presence of the EWS transcript was confirmed by q-RT-PCR. In order to determine early events possibly involved in malignant transformation, we used a multiparameter quantitative strategy that included both MSC immunophenotypic negative/positive markers, and EWS intrinsic phenotypical features. Markers CD105, CD90, CD34 and CD45 were confirmed in EWS samples.

**Results:**

We determined that MSC-P lack the most prevalent gene fusion, EWSR1-FLI1 as well as EWSR1 gene rearrangements. Our study also revealed that MSC-P are more alike to MSC-HD than to EWS cells. Nonetheless, we also observed that EWS cells had a few overlapping features with MSC. As a relevant example, also MSC showed CD99 expression, hallmark of EWS diagnosis. However, we observed that, in contrast to EWS cells, MSC were not sensitive to the inhibition of CD99.

**Conclusions:**

In conclusion, our results suggest that MSC from EWS patients behave like MSC-HD and are phenotypically different from EWS cells, thus raising important questions regarding MSC role in sarcomagenesis.

## Introduction

EWS is a malignant mesenchymal-derived tumor that mainly affects children and young adults. EWS usually arises in bone and soft tissue and is characterized by the presence of a chromosomal translocation between the EWSR1 gene, in the vast majority of cases, and a member of the ETS family of genes, typically FLI1 [1]. This gene fusion encodes a chimerical protein responsible for the transcriptional deregulation of target genes such as the membrane receptor CD99 [1]–[3]. Currently, consensus about the cell of origin of EWS is lacking. This has long been the focus of intensive research and despite recent studies on Neural Crest Stem Cells (NCSC) also suggesting that NCSC can be permissive to EWS fusion, the Mesenchymal Stem Cell (MSC) compartment has been proposed as the most acceptable possibility [1], [4]–[9]. Together with the usual primary location of EWS in mesoderm-derived tissue, *in vitro* and *in vivo* evidence, suggests that MSC may be able to transform into sarcoma-like-cells [10]–[15]. Moreover, EWSR1-FLI1 knockdown shifts gene expression profile from EWS towards an MSC-like signature [16]. Furthermore, the ectopic expression of the EWSR1 fusion in mouse MSC led to tumor development with overlapping features with EWS, namely CD99 overexpression [4]. However these studies were performed in MSC-HD or mouse MSC, while to date MSC-P have never been studied [4], [10], [11], [17]. This fact limits our current knowledge regarding their specific role in sarcomagenesis. More importantly, previous studies performed in MSC derived from cancer patients other than sarcomas, namely multiple myeloma or myelodysplastic syndrome, have shown that in comparison with MSC-HD, MSC from patients exhibit a different genomic or transcriptomic profile [18], [19]. Herein, we characterized MSC-P according to their phenotypical profile and presence of the EWS gene fusion. Our results revealed that MSC-P behave as MSC-HD thus raising important questions regarding their role in EWS sarcomagenesis.

## Materials and Methods

### MSC isolation, expansion and long-term culture

Bone Marrow (BM) samples were harvested by aspiration from the iliac crest from molecularly-confirmed EWS patients under general anesthesia. If the primary tumor was located in the iliac bone, the contra-lateral site was used for BM harvesting (**Table 1**). The ethical committee of the Rizzoli Institute, LUMC and HUSAL approved the studies, and written informed consent was obtained from all subjects involved. BM mononuclear cells were isolated by Ficoll density gradient separation. Washed cells were resuspended in Dulbecco's modified Eagle's-low glucose medium (Gibco, Life Technologies) supplemented with 10% Fetal Bovine Serum (FBS) and Penicillin/Streptomycin (P/S). Cultures were maintained at 37°C in 75 cm^2^ flasks. When cultures reached approximately 70% confluence, cells were detached by treatment with trypsin/EDTA and replated, non-adherent cells were discarded. Cells were stained with trypan blue (Sigma) and counted in a Neubauer chamber. Cells were divided up to a maximum of 6 times. A similar approach was used regarding MSC-HD and MSC from healthy tissues. For full description refer to supplemental data.

**Table 1:** CD99 intensity levels on MSC-P as assessed by Multiparameter Flow Cytometry.

Sample	CD99 MFI^1^	PN 2	Age	Fusion type	Primary tumor	Remarks
**MSC-P-01**	+	P1	12	EWSR1-FLI1	Humerus	**-**
**MSC-P-02**	+	P2	15	EWSR1 BA^3^ positive	Intramuscular	**-**
**MSC-P-03**	+	P3	18	EWSR1-FLI1	Humerus	**-**
**MSC-P-05**	+	P1	17	EWSR1-FLI1	Iliac crest	**-**
**MSC-P-07**	+	P2	20	EWSR1 BA^3^ positive	Fibula	**-**
**MSC-P-08**	+/−	P2	17	EWSR1 BA^3^ positive	Fibula	**-**
**MSC-P-09**	+	P3	24	EWSR1 BA^3^ positive	Rib	**-**
**MSC-P-10**	+	P2	21	EWSR1 BA^3^ positive	Th7	**-**
**MSC-P-11**	+/−	P2	29	EWSR1-FLI1	Iliac crest	MD^4^
**MSC-P-12**	+/−	P3	19	EWSR1 BA^3^ positive	L1 vertebra	MD^4^
**MSC-P-13**	+	P2	31	EWSR1 BA^3^ positive	Humerus	-

All samples were positive for CD99 with low to moderate levels of intensity. Samples were analyzed at low passages (1–3). Primary tumors were located in distinct locations and all presented EWSR1 gene rearrangements and EWS-FLI 1 fusion in some cases. This situation was analyzed with comparison to a control which is the median of all tubes without antibody and intensity levels were calculated with ration between sample/control. (ratio<1− −; ratio around 1 −; ratio = 1, +/−, ratio >2 +, ratio>10 ++, ratio>100 +++).

1 MFI stands for Median Fluorescence Intensity,

2 PN stands for Passage Number;

3 BA stands for Break Apart;

4 MD stands for Metastasis at diagnosis.

### EWS tumor samples

Frozen tissue from EWS samples, (n = 9) were used to assess the expression of CD90, CD105, CD34 and CD45. All H&E and CD99 stained sections were carefully examined by experienced pathologists, confirming the diagnosis.

### EWS cell lines culture

EWS cell lines TC71; RM82; RD-ES; STAET1 and A4573 were cultured in RPMI medium (Gibco) with 10% FBS (Gibco). A673 cell line was cultured in DMEM medium supplemented with 10%FBS. STAET 10, CADO-ES and STAET2.1 were cultured in RPMI medium supplemented with 20%FBS. All media were supplemented with 1% Glutamine (Gibco) and 1% P/S (Gibco). Cell lines have been previously characterized within the EUROBONET consortium [20].

### Multiparameter Flow Cytometry

Cells were collected as described earlier and 2×10^5^ cells were used for each tube.Multiparameter flow cytometry (MFC) immunophenotypic studies were performed with the following monoclonal antibody (MAb) combinations: AF700/AmCyan//PerCPCy5.5/PacificBlue/PE/FITC/APC/PerCPCy7):CD90/CD45/CD34/CD105/CD99, CD166, CD271/CD54, CD106,CD19/CD117,CD73,HLA-DR/CD14, CD13 and CD10 (**Table S1 in File S1**). Cells were stained with the appropriate conjugation of MAb and incubated out of light for 15 minutes. (**Table S2 in File S1**) For CD99 staining, functional studies were performed by indirect immunofluorescence using cloneO13 (Signet, Dedham, MA), as primary antibody diluted 1∶80 and goat anti-mouse FITC (Pierce Biotechnology, Rockford, IL), diluted 1∶100, as secondary antibody.

### Fluorescence in situ hybridization (FISH)

We used a commercial EWSR1 break-apart FISH probe (Abbott-Vysis) for the detection of ES fusion transcripts. Furthermore, an additional, home-made dual-colour FISH probe for the detection of EWSR1-FLI1 rearrangements was developed as follows: three BAC clones (CTD-2307I11, RP11-53D4, CTD-2126E12) spanning the chromosomal region (chr11q24.3) and two BAC clones (RP11-367E7, RP11-480L23) spanning a region on chromosome (chr22q12.1-12.2) were labeled with spectrum green-dUTP (green signal) (Vysis Downers Grove, IL) and spectrum red-dUTP, respectively, by nick translation (Vysis) and purified after adding 10 µg of COT-1 (Invitrogen, Carlsbad, CA). To check the specificity of this probe, hybridizations were performed over metaphases of peripheral blood cells from healthy donors. FISH procedure was performed as previously described, on 2-µm-thick sections [21]. A volume of 10 µl of the diluted probes was applied to the slides. The slide was covered with a glass coverslip and sealed with rubber cement. Using a Hybrite machine (Vysis), denaturation was 75°C for 5 min and hybridization was at 37°C for at least 16 h. After removing the coverslips posthybridization washing was done at 46°C in 2×SSC, 50%formamide for 5 min and stained with DAPI (6-diamidino-2-phenylindole) and mounted with Vectashield H-1000 medium (Vector). Digital images were obtained using a Zeiss Axioplan2 epifluorescence microscope (Carl Zeiss Oberkochen, Germany) equipped with a digital camera (ORCA-ER-1394, Hamamatsu Photonics KK, Hamamatsu, Japan). In all cases, 100 nuclei were counted. TC71 cell line was used as a positive control of EWSR1-FLI 1 rearrangement.

### Differentiation Assays

A minimum of 5×10^4^ MSC were cultured on glass slides for two days in cell culture medium. Afterwards, cells were cultured for 21 additional days in the appropriate differentiation medium, as described elsewhere [18]. For full description refer to supplemental data.

### Immunohistochemistry

Immunohistochemistry (IHC) was carried out on sections of frozen tissue using the Envision method (Dako, CA, USA) and primary antibodies for CD105 and CD90; CD45; CD34 and CD99 (abcam). IHC staining was evaluated by two pathologists

### RNA extraction and qRT-PCR

RNA was extracted using Qiagen RNA mini Kit according to manufacter's instructions. 1 ug of RNA was used to produce cDNA and q-RT-PCR was performed as previously described [24]. The following primers were used for housekeeping gene GAPDH: Forward: GCTCCTCCT GTTCGACAGTCA;Reverse: AATCCGTTGACTCCGACCTTC; and EWS transcripts: i) EWS-FLI-1 type-1 Forward: ATCCTACAGCCAAGCTCCAAGTCA ;EWS-FLI1-type-1 Reverse: ATAAGAAGGGTTCTGCTGCCCGTAG; ii) EWS-FLI-1-type-2 Forward: GATCCTACAGCCAAGCTCCAAGTCA; EWS-FLI-1-type-2 Reverse: GATTGGTGGTGTGGGAGGTTGTAT; iii) EWS-FLI-1-type -3 Forward:GAGAGCGAGGTGGCTTCAAT; EWS-FLI-1-type-3 Reverse: CCCAAGCTCCTCTTCTGACTG and iv) EWS-ERG Forward: CCTACAGCCAAGCTCCAAGTC; EWS-ERG Reverse: GGAAGGAGATGGTTGAGCAG.

### Treatments with anti-CD99 MAbs to evaluate apoptosis-induction

The anti-CD99 0662 MAb was kindly provided by G. Bernard, INSERM 343, Hospital de l'Archet, Nice, France [22]. Here, 2×10^6^ MSC suspended in 200 µl were incubated IMDM 10%FBS with or w/o 10 µg/ml of 0662MAb. After the indicated time-points, control and treated samples were washed and evaluated for Annexin-V–Propidium Iodide evaluation accordingly to the manufacture's instructions (Mebcyto Kit, MBL).

### Data analysis

Hierarchical clustering analysis performed with MFC data was carried out using MSC-P (n = 11), MSC-HD (n = 6), MSC-Adipose derived (n = 1), MSC-Placenta derived (n = 1) and EWS cell lines (n = 9) in which all markers of the previous panel had been tested. Clustering was run using standard correlation coefficients, for the cluster method, a similarity metric and average linkages were used. The Median Fluorescence Intensity (MFI) value obtained for each tested marker was stored into a database and a ratio between the MFI obtained for each marker and the mean MFI value obtained for that marker in all samples tested was then calculated. A table with the percentage of cells analysed for each cell surface marker is shown in Table S2 in File S1. Clustering was performed after median centering and normalizing the fluorescence ratios. A logarithmic (base 2) transformation was applied to the values of this ratio for individual data sets. The resulting normalized log2 ratios were used for further statistical analyses with the J-Express Pro V2.1 software (MolMine AS, Bergen, Norway).

### Statistics

Statistical analysis was performed with PAWS (SPSS Inc, Chicago, IL). U-Mann Whitney test was used to look for significant differences between groups (p values<0.05) and chi-Square test was used to determine significance in the apoptosis assay (significance p<0,05).

## Results

### Characterization of MSC

**Figure 1:**
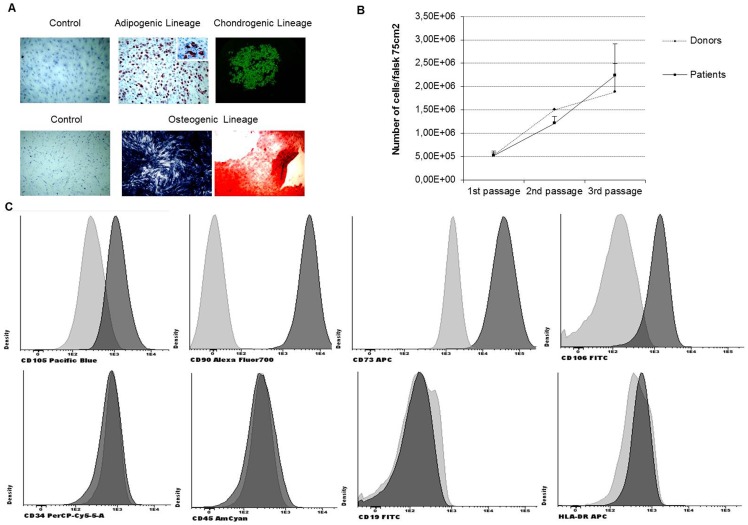
Characterization of MSC. (A) *In vitro* differentiation potential of MSC. *In vitro* adipogenic differentiation. Representative images show the light microscopic view of lipid drop accumulation after 3 weeks of culture in control medium and in differentiation induction medium. *In vitro* chondrogenic potential of MSC. Representative images show the light microscopic view of alkaline phosphatase activity (detected by a blue-dark staining) and mineralization after culture in control medium and in differentiation induction medium (detected by red staining). (B) Growth rate evaluation in MSC-HD and MSC-P. Cells were counted with trypan blue at each passage. Trypan blue selectively colors dead cell in blue whereas live cells are observed shinning since this marker is unable to enter the cytoplasm when the cell membrane in intact. Both sets of samples presented similar growth rates and were in continuous growth during these experiments. (C) Immunophenotypic characterization of MSC: positive expression of CD105, CD90, CD73 and CD106 and negative expression of hematopoietic markers CD34, CD45, CD19 and HLA-DR (light gray represents control and darker gray represents sample with antibody).

All samples were fully characterized according to the International Society for Cellular Therapy (ISCT) regarding i) differentiation potential, ii) plastic adherence and iii) determination of positive/negative surface antigens) [23]. After 21 days of treatment with adipogenic differentiation medium, lipid droplets were observed, confirming their potential to differentiate into adipoblasts (**Figure 1A** **top panel**). MSC were also able to differentiate into osteoblasts which display alkaline phosphatase activity together with calcium deposits (**Figure 1A** **bottom panel**). In addition, MSC showed chondrogenic ability, as confirmed by strong collagen type II positivity (**Figure 1A** **upper-right picture**). All MSC presented plastic adherence. MSC-HD and MSC-P had similar growth rate as observed in **Figure 1B**. Moreover, the samples exhibited a positive expression for the markers: CD105, CD90, CD73 as well as the adhesion molecule CD166. Conversely, the hematopoietic lineage markers CD34, CD45, CD19 and HLA-DR were not found (**Figure 1C**).

### MSC derived from EWS Patients lack EWSR1 gene rearrangements

**Figure 2:**
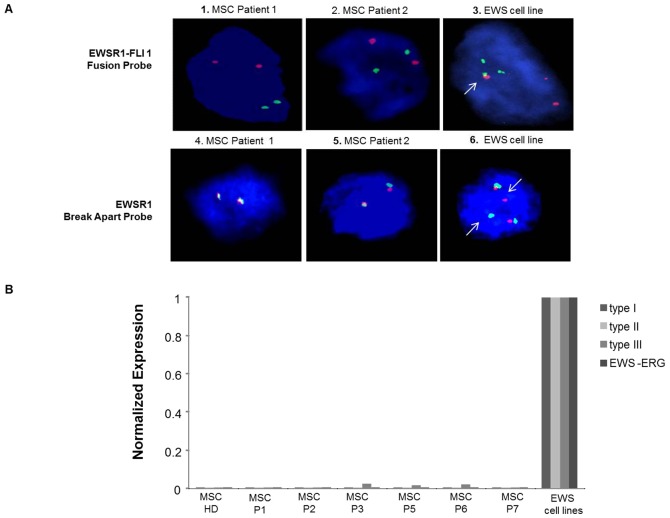
MSC-P lack the presence of the EWSR1-FLI1 chromosomal translocation. (A) Study of the chromosomal translocation between genes EWSR1 and FLI1 using a EWSR1-FLI 1 homemade-fusion probe(1–3). MSC-P showed two normal copies of both EWSR1 and FLI1 (1 and 2) whereas in the TC71 cell line we observed not only two normal copies but also one EWSR1-FLI1 fusion, marked by a white arrow (3) Rearrangements of the EWSR1 gene were studied using a EWSR1 break-apart probe. (4–6) Representative images of MSC-P, EWS-P-01 and EWS-P-02, (4 and 5) failed to present a break in the EWSR1 gene, whereas the positive control here represented by the TC71 cell line (6), showed a distinctive rearrangement of this gene, marked by white arrows. (B) Results were validated by q-RT-PCR using EWS cell lines as a positive control. MSC-P, here represented by Patients 1;2;3;5;6 and 7 as well a healthy donor display a clear negative expression of all the transcripts.

We next explored the presence of the EWSR1-FLI1 gene fusion in MSC-P in all cases. We observed that they lacked this chimeric gene. (**Figure 2A** **, pictures 1 and 2**). Subsequently, we assessed the presence of possible rearrangements of the EWSR1 gene. We observed that MSC-P had no rearrangements of this gene (**Figure 2A** **, pictures 4 and 5**). As expected, the EWS cell lines showed a break on the EWSR1 gene and the fusion gene EWSR1-FLI1 (**Figure 2A** **, pictures 3 and 6, respectively**), therefore these were used as a control. In order to validate this result we also performed qRT-PCR for the various EWS transcripts in BM-hMSC samples. In **Figure 2B**, it may be observed that in comparison to EWS cell lines bearing the different transcripts: TC71 (EWS-FLI-1-type 1; Cado-ES (EWS-ERG); RDES (EWS-FLI1-type 2) and A4573 (EWS-Fli-type 3), MSC-P did not show these transcripts.

### MSC derived from EWS Patients have a low expression of EWS markers

**Figure 3:**
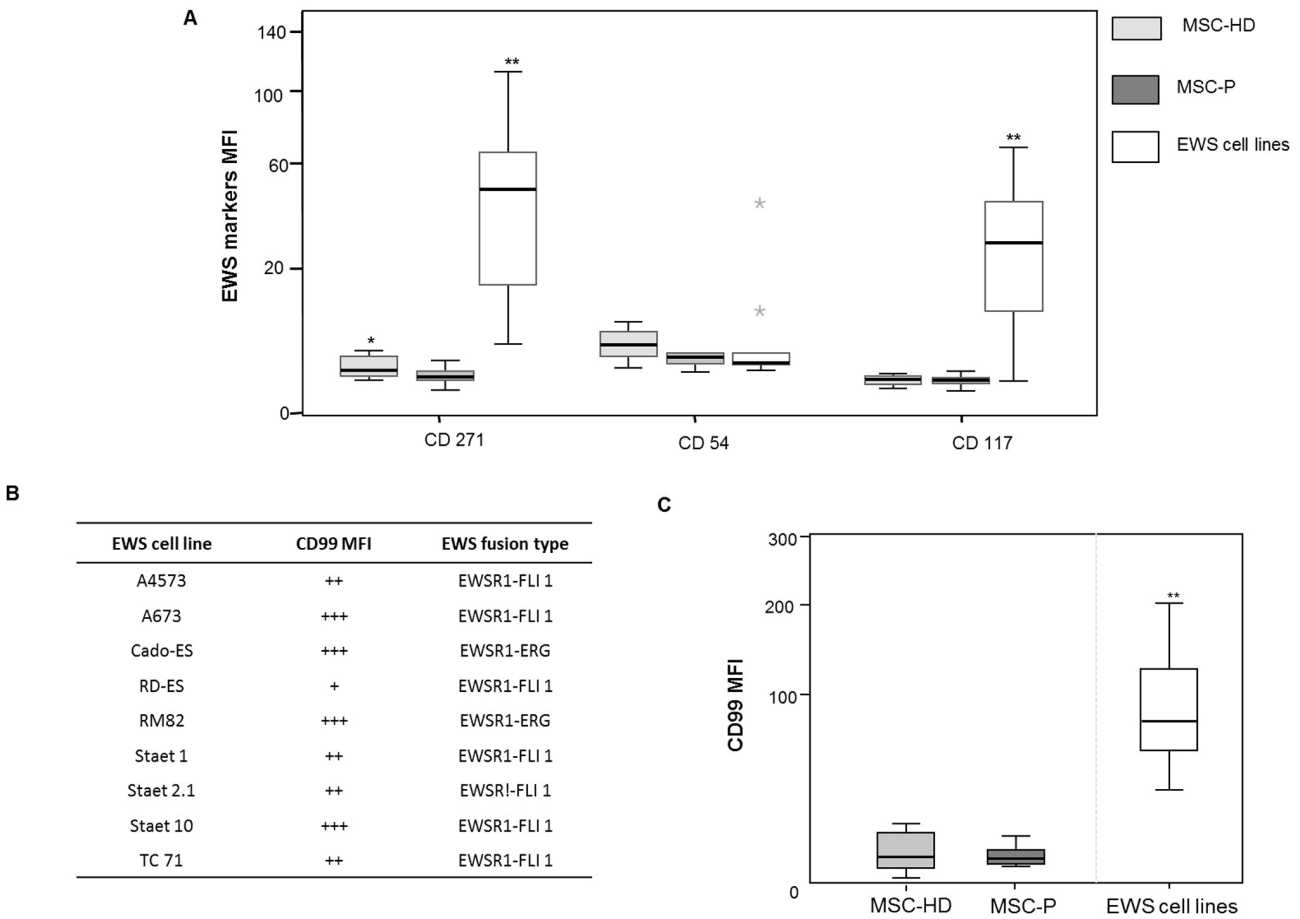
Immunophenotype of MSC-P versus EWS cells. (A) Comparative study between MSC-HD and MSC-P regarding EWS markers: CD271, CD54 and CD117. The expression levels of the EWS markers CD271, CD54 and CD117 were evaluated by MFC and medium values of MFI are represented by groups. (* represent p U-Mann Whitney <0,05 for MSC-P versus MSC-HD and ** p U-Mann Whitney <0,001 for MSC-P versus EWS cell lines). All EWS cell lines present positive expression of CD271, when compared to the control. EWS cell lines show weak expression of CD54 when compared to the control, with the exception of RDES and STAET1 cell lines with strong positive expression values. EWS cell lines present a heterogeneous expression of CD117. (B) Table 1 represents CD99 expression in EWS cell lines. EWS cells presented a rather heterogeneous pattern; nonetheless all cell lines studied here were positive for CD99 expression. Cell lines such as A673 and Cado-ES presented higher levels (score +++) of CD99 expression, whereas RDES cell line presented the lowest levels (score +) of expression. This situation was analyzed with comparison to a control which is the median of all tubes without antibody and intensity levels were calculated with ration between sample/control. (ratio<1− −; ration around 1, − ; ratio = 1, +/−, ratio >2 +, ratio>10 ++, ratio>100 +++). (C) CD99 expression in MSC-P and EWS cell lines. CD99 expression is variable within both groups and similar between the two groups of MSC samples, ranging from weak to moderate, and always lower that the EWS cell lines tested here. (* p Mann-Whitney<0,05 EWS cell lines versus MSC and MSC-P versus EWS cell lines).

The precise immunophenotypic profile of EWS cells has never been fully established. Here, we analyzed the expression of CD271, CD54 and CD117 in a panel of EWS cell lines (**Figure 3A**).

CD271, the low affinity nerve growth factor receptor, displayed a heterogeneous expression pattern among the different EWS cell lines (**Figure 3A**). CD271 has also been reported to be expressed in MSC. In this sense, here we observed a significantly higher expression of CD271 in MSC-HD as compared to MSC-P (p = 0.01) (**Figure 3A**). However, it is worth noting that both MSC-HD and MSC-P presented significantly lower levels of this marker than EWS cell lines (p<0.05) (**Figure 3A**).

CD54 (ICAM-1), an adhesion molecule involved in cell-matrix-cell interactions, was found weakly expressed by all EWS cell lines, with the exception of RDES and STAET 1 (**Figure 3A**). Similarly, CD54 was weakly expressed in MSC, however it was slightly more expressed in MSC-HD compared to MSC-P (**Figure 3A**).

CD117 (C-KIT) has been extensively described as a tyrosine-kinase receptor overexpressed in EWS cell lines and EWS tumor samples [24], [25], [26]. Consistently, most of the EWS cell lines studied here displayed high CD117 expression levels (**Figure 3A**). C-KIT, showed a similar expression in MSC-HD and MSC-P, but its expression was significantly lower than in the EWS cell lines (p = 0.001) (**Figure 3A**).

### The plasma membrane expression pattern of EWS cell lines is globally different from that of MSC

**Figure 4:**
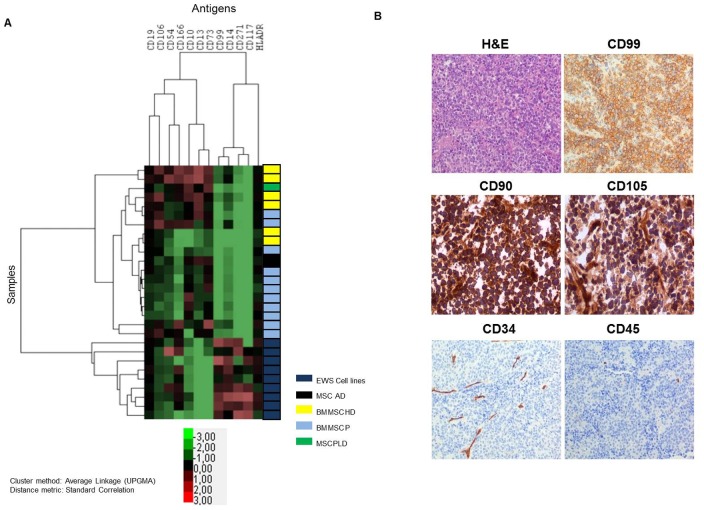
Comparative study between MSC, EWS cell lines and EWS samples. (A) Hierarchical unsupervised cluster analysis showed two different clusters, one consisting of all EWS cell lines and the other of all MSC samples. Control was performed using Fluorescence minus one. The following markers: CD105, CD34, CD45 and CD90 were used in every tube in order to diminish the auto-fluorescence presented by MSC, therefore these were not contemplated in this analysis, and were validated in frozen tissue from EWS samples. (B) IHC studies in frozen tissue from EWs samples. Upper panel represents EWS typical morphology as well as CD99 strong expression. Middle panel shows that a strong expression of CD90 and positivity for CD105. Finally, lower panel shows that EWS present negative expression of both CD45 and CD34.

After studying the presence of EWS markers in MSC, we performed the opposite approach, in which we evaluated the presence of an exhaustive panel of MSC markers in EWS cell lines (n = 9). We performed a hierarchical unsupervised cluster analysis to compare the expression of MSC-positive markers, MSC-negative markers and EWS markers in all samples. We observed a clear division into two clusters, one consisting of all the EWS cell lines and another consisting of all MSC samples (n = 19), which included HD, EWS-P and a set of controls, Adipose and Placenta derived MSC (Figure 4A). Additionally, we evaluated the expression of the base markers CD90, CD105, CD45 and CD34 one by one in a set of EWS samples (n = 9) by IHC. The EWS samples presented the typical small round cell morphology and strong CD99 expression as observed on Figure 4B top panel. CD90 (Thy-1) was strongly expressed in all EWS samples.CD105 (endoglin) was positively expressed in tumor cells and strongly expressed in the cells surrounding the tumor vessels (Figure 4B middle panel). Regarding hematopoietic markers CD34 and CD45, all EWS samples showed negative expression in the tumor cells (Figure 4B lower panel). Results were homogeneously observed in all EWS samples analyzed. Expression of CD90, CD105, CD34 and CD45 in EWS cell lines, in comparison with MSC-HD and MSC-P is detailed on Figure S1.

### MSC derived from EWS patients presented lower levels of CD99 expression than that of EWS cells

CD99 transmembrane glycoprotein overexpression is routinely used as a diagnostic marker of EWS. Herein, we observed that MSC-P presented low to moderate levels of CD99 expression, similarly to MSC-HD and significantly lower than EWS cell lines (p = 0,001) (**Figure 3C**). As depicted in **Table 1**, MSC-P presented comparable intensity levels of CD99 expression between them. Interestingly, both patients MSC-P-05 and MSC-P-11 presented the primary tumor in the same bone from which MSC were collected (iliac bone). Despite being closer to the primary tumor, this MSC presented comparable levels of CD99 intensity to the other patients. MSC-P presented CD99 levels of expression lower than EWS cell lines, with the exception of the RDES cell line, which presented the lowest CD99 intensity. (**Figure 3B**)

### CD99 is heterogeneously expressed in MSC from different tissue sources

We extended our study on CD99 expression to MSC derived from different sources of healthy tissues (BM, iliac crest, amnios, chorion, dental pulp, adipose tissue, and placenta). In this new set of BM-MSC samples, we observed that CD99 expression was also quite variable, ranging from negative to overtly positive (**Table 2**). Regarding MSC from other healthy tissues, all samples with the exception of 2 amnios and 2 adipose derived samples, presented positive CD99 expression (**Table 2**). Overall, weak to highly positive expression of CD99 was found in 17 out of 23 (71%) MSC samples. Of these, at least 11 MSC (scored as ++ and +++) showing expression levels similar to those found in EWS cell lines.

**Table 2:** CD99 expression in MSC derived from healthy tissues.

Sample	CD99 MFI^1^	PN^2^	Localization
**BM-DN-TD**	++	P4	Iliac crest
**BM-DN-HB**	++	P5	Iliac crest
**BM-DN-001**	+++	P6	Iliac crest
**BM-DN-005**	+++	P6	Iliac crest
**BM-DN-HU-1**	+	P4	Iliac crest
**BM-DN-HU-2**	+	P4	Iliac crest
**BM-DN-TD2**	++	P4	Iliac crest
**DP-CM**	++	P5	Dental Pulp
**DP-15**	++	P6	Dental Pulp
**DP-49**	++	P6	Dental Pulp
**COR-3442**	+	P5	Chorion
**COR-3386**	+	P5	Chorion
**COR-3412**	+	P6	Chorion
**AM-3481**	−	P6	Amnios
**AM-3386**	+/−	P6	Amnios
**AM-3396**	−	P5	Amnios
**PLD-2**	++	P4	Placenta
**PLD-3**	++	P4	Placenta
**PLD**	+	P5	Placenta
**AD-HADAS**	+/−	P4	Adipose Tissue
**AD-HADAS-1**	−	P3	Adipose Tissue
**AD-ADIPO3412**	−	P5	Adipose Tissue
**AD-002**	++	P4	Adipose Tissue

CD99 expression levels in MSC derived from normal tissue, including BM; dental pulp; adipose tissue; chorion; amnios and placenta. Again, intensity levels for CD99 expression were variable with 11/19 samples presenting moderate/high expression (scores +++ and ++); 8/19 presenting low levels of expression and 4/19 with negative CD99 expression. Samples were studied between passages 4 and 6.

1 MFI stands for Median Fluorescence Intensity,

2 PN stands for Passage Number.

### CD99 engagement does not elicit impairment of MSC survival

**Figure 5:**
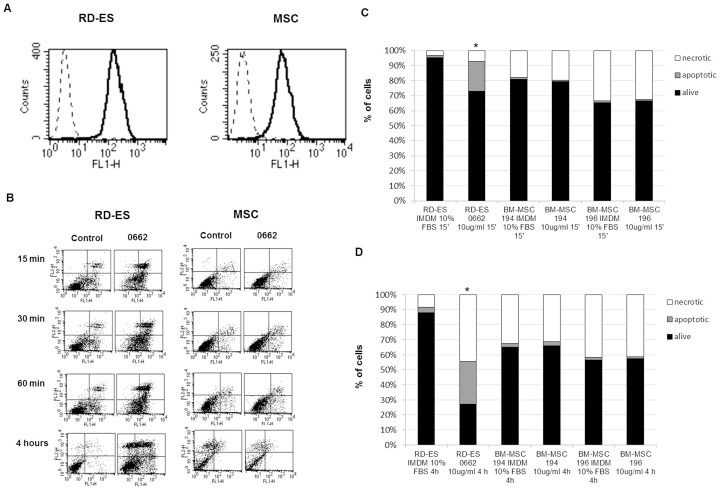
Cytofluorimetric analysis of CD99 expression on RD-ES cell line and BM-MSC. A) Dotted line represents cells stained with secondary antibody alone; solid profile represents cells stained with anti-CD99 antibody. In each panel, the ordinate represents the number of cells. All the 5 MSC used for functional assays presented similar levels of CD99 expression. (B) Time-course analysis of apoptosis after exposure to anti-CD99 0662 MAb. RD-ES is used as positive control. Cell death was determined by examining annexin V-FLUOS binding with flow cytometry. Early-apoptotic cells were annexin V-positive and PI -negative (Lower-Right region). Late apoptotic/necrotic cells were annexin V- and PI-positive (Upper-Right region). Results shown here are representative of all the 5 CD99-highly positive MSC. (C) Percentage of living, apoptotic and necrotic cells observed in cells (two representative cultures of BM-MSC and RD-ES EWS cell line) treated with anti-CD99 MAb for 15 min or (* represents statistical significance in comparison to control p<0,05)(D) 4 hrs. The assay was performed on detached cells. The EWS cell line shows an increment in the percentage of apoptotic and necrotic cells after CD99 engagement, whereas the BM-MSC show similar percentages of alive and dying cells in treated or untreated conditions. (* represents statistical significance in comparison to control p<0,05).

We evaluated whether engagement of CD99 with 0662 MAb was able to induce apoptosis as previously shown in EWS cells and MSC (**Figure 5A**) [27], [28]. Cell cultures from different BM-MSC (n = 5) positive for CD99 at similar levels, were exposed to anti-CD99 MAb and RDES was used as a control (**Figure 5B**). We observed that a mere 15 minute-treatment was enough to induce a 27% of apoptotic cells in RDES (p<0.05), whereas the same treatment regimen only triggered a 7% of apoptotic population in MSC (**Figure 5C**). Furthermore, 4 hours of treatment lead to an almost complete apoptotic RDES culture (73% annexin-V positive cells) (p<0.05) but failed to increase significantly the initial percentage of apoptosis in MSC (**Figure 5D**).

## Discussion

Previous evidence suggesting that MSC would be the most plausible cell of origin of EWS have stressed the need for deeper studies of BM-MSC derived from EWS patients. Clinically, the diagnosis of EWS is based on morphology, by the presence of the EWSR1-ETS fusions, and the cell surface over expression of CD99 [1]. The primary aim of our study was to determine early events in MSC-P, such as the presence of EWSR1-ETS fusions and differential phenotypic patterns, as a way to unveil cellular predispositions that could be exploited in therapy. Specifically; we observed that MSC-P lacked the presence of the EWSR1-FLI 1 fusion gene and further EWSR1 rearrangements. These results were validated at the DNA and mRNA level. This might not be surprising, but it is important given that previous studies performed in Leukemia described that fusion of the MLL (HRX, ALL-1) gene with its partners, initially arises in prenatal myeloid cells, which are pre-malignant, which after stages of progression during development result in fully malignant leukemic-cells in the adult [29].

Subsequently we analyzed the presence of EWS surface markers. Our goal was to determine events occurring prior to the translocation that could enable a permissive environment for cell transformation and perhaps help solve the causal dilemma of what came first, the EWSR1 fusion or secondary alterations: the chicken or the egg? [30]. Our results, however, suggest that the immunophenotypic profiles of MSC-P and MSC-HD, are similar regarding EWS surface markers CD99, CD271, CD54 and CD117. Notwithstanding, our study also revealed that both EWS cell lines and EWS samples display MSC features such as the expression of CD90 and CD105 and the lack of the hematopoietic markers. The absence of CD34 expression in EWS cell lines and samples, is in fact very interesting, given that autologous stem cell transplantation has already been considered in clinical trials for EWS [31]. Our results rule out the possibility of contamination of the CD34+ niche with tumor cells in auto transplantation.

In line of the above, we cannot discard the possibility of a cellular hierarchy between MSC expressing CD99; cellular pre-malignant stages presenting EWSR1 gene rearrangements and gene deregulation, gradually losing MSC phenotype and; finally a fully malignant EWS cell bearing the EWSR1 fusion, overexpressing CD99 and maintaining some MSC phenotypical characteristics such as CD90 and CD105.

Since MSC also express CD99, and taking into account that CD99 is a promising target for innovative therapies in EWS, we felt the need to study possible cytotoxic effects of anti-CD99 therapies on MSC. CD99 is, in fact, overexpressed in over 99% of EWS cells and has been considered as a key causative of EWS malignancy and a potential therapeutic target [1], [3]. Contradictory data suggest that on the one hand, induced expression of EWSR1-FLI1 upregulates CD99. On the other hand, however, when EWSR1-FLI1 was knocked down in EWS no changes in CD99 expression were reported [6], [11], [32].These observations may actually be reconclied by our finding that MSC show variable baseline expression levels of CD99. The physiological functionality of CD99 remains unknown, but CD99 knockdown in EWS cells induced their neural differentiation and reduced cell malignancy [33]. Altogether, our previous data confirmed that both EWSR1-FLI1 and CD99 are absolutely critical for EWS oncogenic phenotype maintenance [33]. Additionally, in EWS, the engagement of CD99 with antibodies resulted in apoptosis of EWS cells [34]–[36]. Here, we observed that MSC displaying similar CD99 expression levels to EWS cells were unable to undergo apoptosis when exposed to 0662. This finding is of particular interest in terms of future clinical application of CD99 targeted therapies, since it sustains the limited toxicity to MSC and potentially high specificity of this therapeutic approach in EWS patients. However, toxicity towards other cell types expressing CD99, should also be tested.

In conclusion, i) MSC-P behave like MSC-HD and are phenotypically different from EWS cells; ii) CD99 is variably expressed among MSC derived from normal tissues, and finally iii) anti-CD99 therapies induce massive cell death in EWS cells while maintaining viability and integrity of MSC with similar CD99 expression.

## Supporting Information

Figure S1 **MFI of markers CD90, CD105, CD34 and CD45 in MSC-HD, MSC-P and EWS cell lines.**
(TIF)

File S1 **Tables S1 and S2.** Table S1. Mab antibodies used in the multiparametric analysis and functional assays. Table S2. Percentage of Cells evaluated for each cell surface marker.
(DOC)
