# Correction: Characterization of Human Mesenchymal Stem Cells from Ewing Sarcoma Patients. Pathogenetic Implications

**DOI:** 10.1371/journal.pone.0094455

**Published:** 2014-04-03

**Authors:** The PLOS ONE Staff

In the Funding statement, multiple funding organizations and grants to multiple authors are incorrectly omitted. The Funding statement should read:

Research in Enrique de Alava's lab is supported by the Ministry of Science and Innovation of Spain-FEDER (PI081828, RD06/0020/0059, PI110018, ISCIII, postdoc grant CD06/00001 to JLOG), the Fundação para a Ciência e Tecnologia, Ministério para a Investigação e Tecnologia, Portugal (ATA fellowship SFRH/BD/69318/2010) and the Red Temática de investigación del cáncer (RTICC, Spain). This work was also supported by the European Commission (FP7-HEALTH-2011-two-stage, Project ID 278742 EUROSARC). Katia Scotlandi's lab is also funded by AIRC, Italian Association for Cancer Research, (project 10452 to KS), Progetto FIRB- Accordi di programma 2010 COD, RBAP10447, and by the Italian Ministry of Health (Project IOR-2006-422755). The funders had no role in study design, data collection and analysis, decision to publish, or preparation of the manuscript.
