# Supplementary figures and images for: Characterization of Human Mesenchymal Stem Cells from Ewing Sarcoma Patients. Pathogenetic Implications
Source: PLoS One. 2014 Feb 3;9(2):e85814. doi: 10.1371/journal.pone.0085814 (PMC3911896; doi:10.1371/journal.pone.0085814)

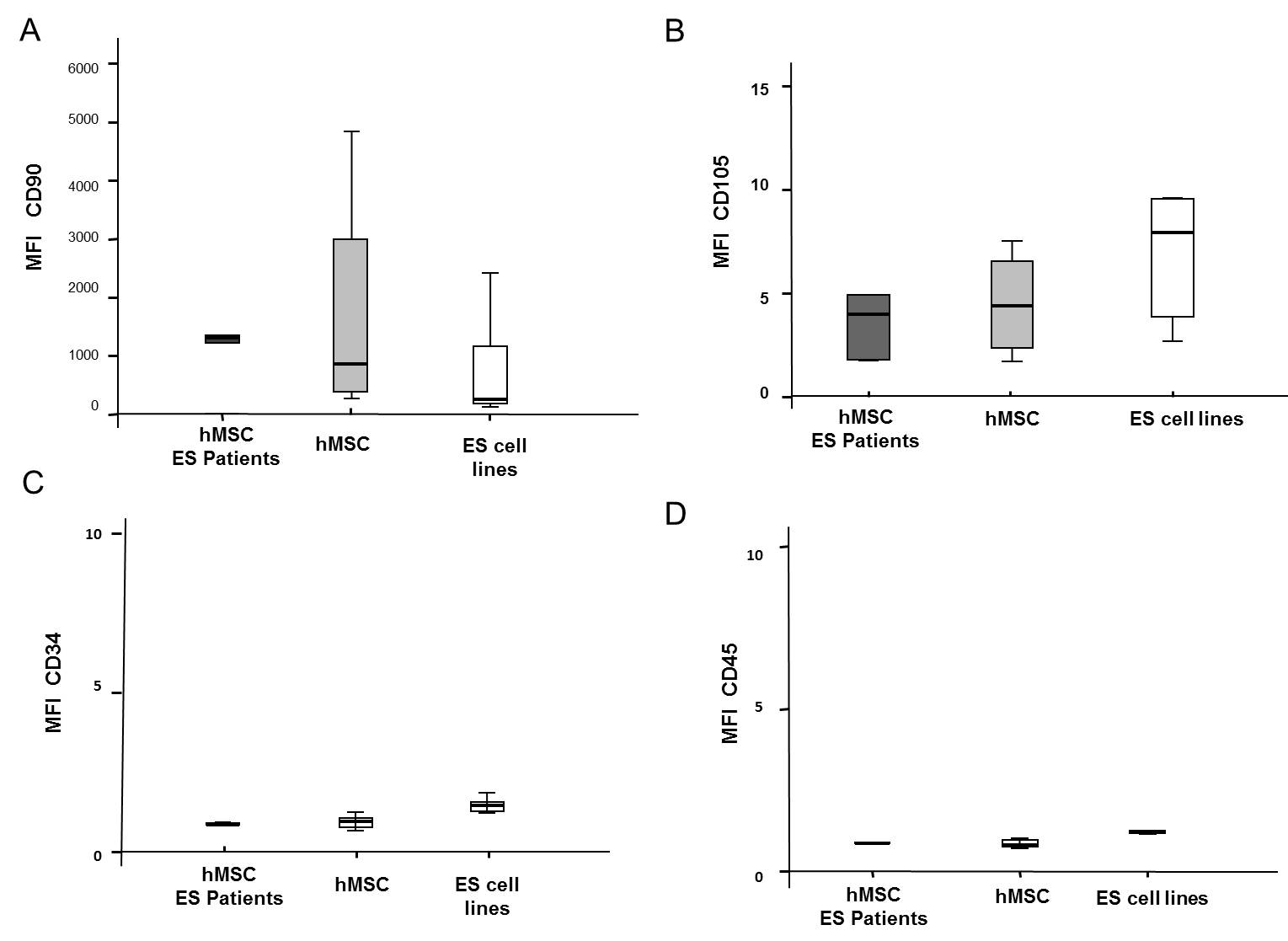

Supplement: Figure S1 — MFI of markers CD90, CD105, CD34 and CD45 in MSC-HD, MSC-P and EWS cell lines. (TIF) [file pone.0085814.s001.tif]
