# Supplementary material for: Characterization of Human Mesenchymal Stem Cells from Ewing Sarcoma Patients. Pathogenetic Implications
Source: PLoS One. 2014 Feb 3;9(2):e85814. doi: 10.1371/journal.pone.0085814 (PMC3911896; doi:10.1371/journal.pone.0085814)
Supplement: File S1 — Tables S1 and S2. Table S1. Mab antibodies used in the multiparametric analysis and functional assays. Table S2. Percentage of Cells evaluated for each cell surface marker. (DOC) [file pone.0085814.s002.doc]

**File S1**

**Supporting Information**

**Supplementary Material and Methods**

**MSC samples description**

BM-MSC-P (n=11) were provided by LUMC (Leiden University Medical Centre, The Netherlands), whereas BM-MSC-HD (n=6) were provided by the HUSAL (University Hospital of Salamanca, Spain). In addition, MSC derived from other sources were also considered. MSC derived from adipose tissue (n=4) from IOR (Istituto Ortopedico Rizzoli, Bologna, Italy) and HUSAL, Placenta derived MSC (n=3) from HUSAL, dental pulp (n=3) amniotic membrane (n=3) and chorion membrane (n=3) from the University of Bologna (Bologna, Italy) were isolated and expanded as previously described (18-22). The ethical committee of the Rizzoli Institute, LUMC and HUSAL approved the studies, and informed consent was obtained from all subjects involved.In the first approach, MSC from low passages, between 1 and 4, were fully characterized at the immunophenotypic level. These include MSC from EWS-P(n=11); MSC-HD (n=6); MSC derived from adipose tissue (n=1) and MSC derived from placenta (n=1). In the second approach, including MSC from higher passages (n=14), these were used only for CD99 detection, due to the determinant number of cells necessary to perform the full study.

**Differentiation Assays**

Briefly, regarding osteogenic differentiation, cells were cultured in Alpha-MEM (Invitrogen) supplemented with 10% FBS; 1% P/S; 0,1μM Dexamethasone (Sigma Aldrich); 10mM Glycerol-2-Phosphate (Sigma) and 50μM 2-Phospho-L-Ascorbic-Acid (Biochemika) and later on stained for alkaline phosphatase activity detected with BT-BCIP and for calcium deposits with Alizarin Red. Adipogenic differentiation was evaluated after incubation in Alpha-MEM (Gibco) medium supplemented with 10%FBS; 0,1μM Dexamethasone (Sigma); 50μg/ml Human Insulin (Sigma); 0,5mM Isobutylmethylxantine (Sigma) and 60μM Indomethacin (Biochemika). Lipid drop formation was evaluated with Oil Red staining. To induce chondrogenic differentiation, aliquots of 2.5x10^5^ cells were pelleted in polypropylene conical tubes in 0.5 ml of differentiation medium as previously described (16). Pellets were formalin-fixed, embedded in paraffin, examined morphologically and immunostained for Type II collagen (Chemicon Int, Tamecula, CA).

**Supplementary Figure captures and Tables**

**Table S1. Mab antibodies used in the multiparametric analysis and functional assays.**

**Table S2. Percentage of Cells evaluated for each cell surface marker**

**Figure 1S. MFI of markers CD90, CD105, CD34 and CD45 in MSC-HD, MSC-P and EWS cell lines.**

Table S1

| **Antibody** | **Dilution** | **Commercial Source** |
| --- | --- | --- |
| CD90-AF700 | 1:100 | Biolegend |
| CD45-AmCyan | 4:100 | Becton Dickinson, BioSciences |
| CD34-PerCP | 10:100 | Becton Dickinson |
| CD105-PB | 4:100 | Exbio |
| CD99-PE | 10:100 | BD Pharmingen |
| CD166-PE | 10:100 | Beckman Coulter |
| CD271-PE | 10:100 | Becton Dickinson |
| CD54-FITC | 10:100 | ExBio |
| CD106-FITC | 10:100 | BD Pharmingen |
| CD19-FITC | 10:100 | Becton Dickinson |
| CD117-APC | 3:100 | Becton Dickinson |
| CD73-APC | 3:100 | eBioscience |
| HLA DR-APC | 3:100 | Becton Dickinson |
| CD14-PeCy7 | 5:100 | Becton Dickinson |
| CD13-PeCy7 | 5:100 | Becton Dickinson |
| CD10-PeCy7 | 5:100 | Becton Dickinson |
| CD99 | 1:80 | Covance, Princeton,NJ |

**Table S2**

| **Ewing Sarcoma Cell Lines** | | | | | | | | | | | | | | | | | | |
| --- | --- | --- | --- | --- | --- | --- | --- | --- | --- | --- | --- | --- | --- | --- | --- | --- | --- | --- |
|  | **A4573** | | **A673** | | **CADO-ES** | | **RD-ES** | | **RM-82** | | **STAET10** | | **STAET1** | | **STAET2.1** | | **TC71** | |
|  | % | +/- | % | +/- | % | +/- | % | +/- | % | +/- | % | +/- | % | +/- | % | +/- | % | +/- |
| CD19 | 0% | - | 0% | - | 0% | - | 0% | - | 0% | - | 0% | - | 0% | - | 0% | - | 0% | - |
| CD271 | 100% | ++ | 100% | ++ | 100% | ++ | 100% | ++ | 100% | ++ | 100% | ++ | 100% | ++ | 100% | + | 100% | + |
| CD34 | 0% | - | 0% | - | 0% | - | 0% | - | 0% | - | 0% | - | 0% | - | 0% | - | 0% | - |
| CD10 | 0% | - | 0% | - | 0% | - | 0% | - | 0% | - | 0% | -- | 0% | - | 0% | - | 0% | - |
| HLA-DR | 0% | - | 0% | - | 0% | - | 0% | - | 0% | - | 0% | - | 0% | - | 0% | - | 0% | - |
| CD90 | 100% | ++ | 100% | ++ | 100% | ++ | 100% | ++ | 100% | ++ | 100% | ++ | 100% | ++ | 100% | ++ | 28% | -/+ |
| CD105 | 100% | + | 100% | + | 100% | + | 100% | + | 100% | + | 100% | + | 100% | + | 100% | + | 100% | + |
| CD45 | 0% | - | 0% | - | 0% | - | 0% | - | 0% | - | 0% | - | 0% | - | 0% | - | 0% | - |
| CD106 | 0% |  | 0% | - | 0% | - | 0% | - | 0% | - | 0% | - | 0% | - | 0% | - | 0% | - |
| CD166 | 100% | ++ | 100% | ++ | 0% | - | 100% | ++ | 100% | ++ | 100% | ++ | 100% | ++ | 100% | + | 100% | + |
| CD13 | 0% | - | 0% | - | 0% | - | 30% | -/+ | 0% | - | 0% | - | 0% | - | 0% | - | 0% | - |
| CD73 | 0% | - | 100% | + | 0% | - | 56% | -/+ | 0% | - | 0% | - | 0% | - | 0% | - | 100% | + |
| CD54 | 0% | - | 0% | - | 0% | - | 100% | ++ | 0% | - | 0% | - | 100% | + | 0% | - | 0% | - |
| CD99 | 100% | ++ | 100% | +++ | 100% | +++ | 100% | + | 100% | +++ | 100% | +++ | 100% | ++ | 100% | ++ | 100% | ++ |
| CD14 | 80% | + | 88% | + | 100% | + | 0% | - | 0% | - | 0% | - | 0% | - | 0% | - | 0% | - |
| CD117 | 100% | + | 0% | - | 100% | + | 38% | -/+ | 100% | ++ | 100% | + | 100% | + | 100% | + | 0% | - |
